# Medicine among the Mormons and the Indians of North America

**Published:** 1862-02

**Authors:** 


### MEDICINE AMONG THE MORMONS AND THE INDIANS OF NORTH AMERICA.

It may be remembered that in the Autumn of 1858, the United States sent a military expedition to Utah, against the Mormons. The Reports* of the Surgeons who accompanied

* "Statistical Report on the Sickness and Mortality in the United States Army, from 1855 to 1860." Washington.

the expedition and saw something of Mormon life, are of great value, as showing the effects which complete isolation and gross religion, whose very essence is Polygamy, have produced on the physical stamina and mental health of that saintly community.

Among the males, sexual debility, with its concomitant evils, is stated to be the most common result of this premature stimulation of the sexual passion, and the facilities of gratifying it. Among the women these same causes show their pernicious effects, not in diminution of generative power, but in physical deterioration of the offspring. The women are even remarkable for their fecundity, but their children are ill-nourished and cathetic, a large proportion of them dying in infancy, and those who *do* live to maturity, looking feeble, sickly, stunted, and (the males) emasculate. Mormonism appears to have made its impress on the countenance in a remarkable manner. "There is an expression of countenance," says one of the Surgeons, "and a style of feature which may be called the Mormon expression and style—an expression compounded of sensuality, cunning, suspicion, and smirking self-conceit. The yellow, sunken, cadaverous visage; the greenish-colored eyes; the thick protuberant lips; the low forehead; the light, yellowish hair; the lank, angular person, constitute an appearance so characteristic of the new race, the production of polygamy, as to distinguish them at a glance. The older men and women present all the physical peculiarities of the nationalities to which they belong; but these peculiarities are not propagated and continued in the new race; they are lost in the prevailing Mormon type."

The Mormon faith inculcates that diseases can be cured only by miraculous interposition; consequently, the secular professors of the healing art are not much patronized. "The Church authorities are exceedingly jealous at an attempt to cure by ordinary therapeutics, and denounce from the pulpit any invasion of their special province. Though they claim for the laying on of hands wonderful efficacy, the number of deformities—the result of malpractice, to be seen in any of the populous towns—rather indicates a necessity for the use of carnal means. The art of surgery is at a low ebb."

The only disease peculiar to the Utah Territory is what is called in the reports "Mountain Fever." The minute descriptions given of this disease leave no doubt in our mind that it is nothing more than a form of periodical fever, modified by elevation and other hygienic conditions. The persons who most suffer from it are the trappers and Indians when following the rivers to their mountain sources for the purpose of trapping beaver. With this exception, no diseases of a severe character prevail in the Territory, the climate of which is unrivalled for healthiness. In the different reports much uncertainty is expressed whether this "mountain" fever owns the same cause as ordinary intermittent fevers; but when we read the topographical descriptions of the localities where it prevails and the seasons to which its prevalence appears to be restricted, we cannot for a moment doubt that both kinds are attributable to the same, and to no other, efficient cause, viz: marshy ground in the process of drying under the influence of solar heat.

The reports of the Army Surgeons, located at the various military posts in Utah, state that the climate of that country exercises singularly beneficial effect upon phthisis. They were stationed in that Territory from the autumn of 1857 to the spring of 1859, a sojourn long enough to render their observations, both on this and on other matters, worthy of our credit.

The Utah Territory has an average elevation of about 6000 feet above sea level. It consists of ranges of hills, with intervening broad table lands, valleys and streams. What renders its climate remarkable is its *equability and dryness.* Although strong south-westerly winds are almost constantly prevalent, and the range of temperature in the course of the year is considerable (from about 90 deg. in summer to about 20 deg. in winter), *sudden vicissitudes* of temperature are very rare; the summers being regularly hot and the winters regularly cold. The absence of moisture is indicated by the hygrometer and by the freedom of arms and surgical instruments from the slightest traces of rust. The great dryness is stated to render the extreme cold much less appreciable to the senses, so that a smaller amount of clothing is necessary than in latitudes warmer but moist. Such being the nature of the climate, the reports show that the prevalence and mortality of phthisis are much less at the Utah station than at any other military district in North America. In 1858, out of 5,842 men stationed in Utah, only eight cases of phthisis occurred, and only one of these proved fatal. The freedom from pulmonary complaints of *all* kinds in this country, is equally remarkable. That this comparative immunity from phthisis, and lung disease generally, is due rather to peculiarity of climate than mode of life, seems clear from the fact that those, on whom the observations have been made, are soldiers—a class of men whose habits are much the same everywhere. So notorious is the healthiness of these high regions of the far West, that the subjects of hereditary or acquired predisposition to phthisis or scrofula, as well as other invalids, have for some time past been largely migrating to them, as being the climate of all others in North America the most suitable for their maladies. The journey across the plains from the Atlantic States is not now nearly so formidable an enterprise, as it was a few years back. Along the great roads constituting the overland route to California, the journey to the West can now be accomplished without danger or delay. So much for Utah, its inhabitants, diseases and climate. We will now turn to some of the adjacent Indian tribes of North America, concerning which we find several interesting details in the reports before us.

Intemperance and disease, especially syphilis, scrofulas phthisis, small-pox, cholera and fever—coupled with the evil, incident to a state of almost perpetual warfare—are said to be slowly exterminating the aboriginal inhabitants of North America. Another influence, tending in the same direction, is the steady advance of civilization, the restraints of which are incompatible with the nature and habits of these children of the forest. As the Indians will not blend with civilization, they must recede and dwindle away before its onward progress. The Indian, when his natural habits are interfered with, "feels himself a prisoner and deprived of his dearest rights; his spirit is broken, and with the change of diet and wearing apparel, and being deprived in great measure of his natural exercise, disease takes hold of him and he sinks under it. As a general thing, deprive an Indian of his accustomed exercise and give him plenty of beef and flour, he gets dysentery; and when he changes his wearing apparel, laying aside the open blanket and putting on the white man's tight coat, and lies down to sleep, his lungs take on disease, and soon become full of tubercles." Such is the testimony of the American Surgeons who have lived among them and closely watched their ways.

The Indian tribes are said to have no regular physicians among them. The "Medicine man" is a sort of half priest, half doctor, who possesses mysterious remedies, and is regarded with superstitious awe. Among many of the tribes, especially those on the frontiers of civilization, there are swarms of traveling charlatans and heartless quacks, who ply their trade with success proportioned to the unbounded credulity of the buyers of their vile nostrums. Phthisical patients are generally left to their fate. Phthisis is one of their commonest diseases, and as it is regarded by the majority of the Indians as communicable by contagion, especially among the family of the afflicted individual, the poor sufferer has to drag out the most miserable solitary existence, till death relieves his sufferings. Barring the use of a few inert remedies of a domestic nature, diseases in the majority of cases is left to run its course. Some tribes, on the other hand, adopt methods of treatment altogether the reverse of the expectant. The New Mexican Indians, for instance, suffer much at certain seasons of the year from intermittent fever; and for the cure and prevention of this disease, large numbers of them assemble once a year (in the spring), and for the space of three day swallow frequent draughts of the infusion of some powerful herb, which causes violent vomiting and purging. This is called a "Medicine Feast," and is supposed to cure the fever where it already exists, and to fortify the system against its attack during the ensuing summer and autumn. Whether as a curative or as a prophylactic measure it is attended with any success, is not stated.

The New Mexican Indians have another annual gathering, a "feast" of a more truly festive description than the one just mentioned. It takes place in the winter, on the mountains, when they "distil a nasty, filthy kind of liquor from corn, which is known among them as *tiswing*, of which they are passionately fond, and never lose an opportunity to get gloriously drunk, whenever a little corn can be procured to make it from. Their mode of distillation is as novel as it is simple. The corn, procured by stealth, or as a 'forced loan,' is steeped for twenty-four hours, then placed between the scanty folds of of the family couch to ferment* beneath the bodies of the lusty warriors, whose slumbers are soothed by bright dreams of 'the good time coming,' when all shall be assembled at the festive wigwam to partake of the intoxicating pleasures of this unpretending beverage. The malt is now coarsely ground, a certain amount of water added to it, then placed in earthen vessels and allowed to ferment for forty-eight hours, at the end of which it is used without straining or any other process. The better to enjoy 'the feast of reason and the flow of soul,' these votaries of Bacchus prepare themselves by a solemn fast of forty-eight hours previous to the commencement of the festal

*The writer evidently means "to germinate."

joys, for the purpose of enjoying the most profound spiritual impressions—in other words—they get 'right royally drunk,' on empty stomachs. Were it not for this precaution, the mild character of the beverage would fail 'to make the drunk come.' "

The Oregon and many other Indians have one chief remedy for all internal diseases. This consists of a hot vapor-bath followed immediately by a plunge into a cold stream. Such is their mode of treating small-pox, for instance; the result in almost every case is stated to be fatal.

The North Californian Indians treat disease in a manner totally different from that of the Indians east of the Rocky Mountains. They resort neither to the animal, nor vegetable, nor the mineral world for a cure. "Their philosophy of disease is based upon the idea that an evil spirit of some dead Indian steals into the body and locates himself, and wherever the pain may be, there the spirit is. Their system of therapeutics is perfectly compatible with their notions of pathology. Their doctors are always females, and none are called to practice except those who are commissioned as it were by nature. When a young squaw chances to have a slight periodical hæmorrhage from the mouth or any other part than the natural channel—in other words, if she should have vicarious menstruation,—she is the one who for ever after is destined to heal the sick. The young squaw is not at all covetous of her calling; she dreads her profession, for on her failure to effect a cure, she loses her life. Her art of medication consists in working herself up into a mesmeric or hysterical condition, and then making passes over the part diseased, and lastly plunging her fists into the muscles of her prostrate patient, as if she were really determined to tear out and expel the dead Indian's evil spirit in good earnest. Then, again, the doctress throws herself into a gladiatorial position, being surrounded by a dozen or more 'bucks,' who assist in the performance by chanting and howling like so many demons; and then she throws herself with her whole weight on her subject, clinching the part diseased, and if she is lucky she drags out the evil spirit (with a portion of the patient's skin,) and dashes it into a vessel of water ready for that purpose. If the patient is satisfied that he is relieved of the dead Indian's spirit and the pain, all is right; but if the pain chances to return, the poor squaw loses her head under the tomahawk."

The climate of California would appear to be singularly healthy and invigorating. In one of the Medical Reports for that country, it is mentioned as a notorious fact, that when females from the Atlantic, or from the inland States, go to live in California, their uterine functions assume a greatly increased activity; that menstruation becomes much more copious; that those who have been barren for years, and those who have never been pregnant at all, after a short residence in their new climate, are almost certain to become "joyful mothers of children." The veneral propensities of the other sex are said to derive a fresh impulse under the same circumstances. It is further stated, as a matter of fact, that the fecundity of cows, sheep and pigs, greatly increases, if they are brought from the plains of the interior to the Californian coast. We should receive such a statement with great distrust, were it not made in such an unhesitating and unqualified manner by a medical gentleman in the United States Army, whose residence in the country must have given him abundant opportunities for investigating the truth of the matter.

Child-bearing among the Indians is stated to present the same delays and accidents as among the civilized inhabitants. In tedious labor pressure is made on the abdomen with the hand, and, if this does not expite matters, a hoop is placed round the waist, and forced downwards. Hence we are not surprised to hear that prolapsus uteri is no common among them.

Syphilis is exceedingly rife among the Indian tribes, but more so in the southern than the northern parts of the continent. Two reasons are assigned for this in the report: 1. From the middle of the sixteenth century up to the present time, the Spaniards have been mixed up with the aborigenes, and have freely sown the seeds of the disease among them. 2. The Spaniards seldom visit the northern parts, and if they do, the colder and purer climate renders the virus less infectious and more likely to give way under the influence of natural causes. The northern and central tribes believe the disease to have been disseminated among them by the Hudson Bay traders; not under the natural impulses of sexual passion, but with the "pale-faced," cold-blooded design of undermining their strength and thinning their numbers. Scoundrels as many of these traders undoubtedly were, one is loth to impute to them such villainy as this.

So terrible have been the ravages of syphilis, that the Indians of New Mexico have had the wisdom to adopt penal measures, which are found greatly to check the spread of that disease. Any member of their tribes, who is known to have contracted the disorder, is either tomahawked forthwith, or, like the leper of old, excluded from all intercourse with his cleaner fellowmen, and abandoned generally to die of starvation. Dread of a puuishment, so certain and so terrible, makes them cautious about exposing themselves to the chances of infection.

The following novel mode of treating retention of urine, is related in one of the reports:—In 1857 an unfortunate half-bred Frenchman applied to the Surgeon at Fort Randall, in Nebraska, for the relief of stricture of his urethra, which had been caused by gonorhœa contracted twelve months previously. He had suffered several bad attacks of retention, and on one occasion had been obliged to have recourse to the skill of an Indian doctor, whose mode of treatment, whatever may be said against it, possessed, at least, the triple merit of novelty, simplicity and success. The doctor applied his lips to his patient's penis, and by repeated vigorous efforts at suction, drawing off a *bon-bouche* at a time, managed at last completely to relieve the enormously distended bladder. It had been previously stipulated that the poor Frenchman, as a consideration for the service for the service performed, was to give the doctor his only horse. We have heard of the Hindoo surgeons using suction for extracting a cataract after incising the cornea; but the above application of nature's suction-pump for the relief of suffering humanity struck us an idea truly original, though we have since seen, in an old number of the *Gentleman's Magazine*, that an English surgeon performed the same officer for Fernandino, one of the Earls of Derby, in the seventeenth century. We place the hint at the disposal of the profession. The ingenuity of some enterprising surgical mechanist may, perhaps, turn it to account and reproduce it in some more palatable form, such as the exhausted glass air-chamber used by Sir Philip Crampton, to remove fragments of broken calculi with viscid mucus from the bladder after lithotrity, or the powerful syringe we have ourselves used for the same purpose.—*London Medical Times and Gazette.*

